# Boston Letter

**Published:** 1902-12

**Authors:** 


### Domestic Correspondence.

### BOSTON LETTER.

To the Editor:

Sir,—Vacations are over and the fall work has begun in earnest. Every one appears in excellent spirits after their well-earned rest. Many of our friends have taken long and delightful trips. Dr. Julius Werner spent the summer in Europe, Dr. Clapp was at his Maine camp for a long stay, others have had equally pleasant times, and generally most every one has given up some time to the study of geography and tried to forget all about where they came from.

The Harvard Odontological Society held its first meeting after the summer vacation at Young's Hotel, September 25. For some years its meetings have been held at that popular hostelry, but now owing to various changes the club will no longer meet there, its next meeting being booked for the Hotel Westminster.

The speaker of the evening was Dr. Clarence B. Vaughan, who read a paper on the bleaching of teeth. Dr. Vaughan covered the ground carefully but concisely, and his paper and the discussion which followed were of great value.

Dr. H. H. Stoddard showed a system of porcelain inlays as advocated by Dr. Peck, and passed about many samples of the work and also Dr. Peck's book and system.

The meeting adjourned at ten P.M., with expressions of satisfaction and good fellowship from all sides. A number of the men have been to the State dental meeting at Worcester this week, and report that it was one of the best ever held.

The Harvard Dental School is also in full swing. The number of men entering is smaller than usual, owing to the increased severity of the examinations, but it can be seen at a glance how much this has raised the standard of the school. The men are older and appear much more in earnest than ever. They promise well for the future of dentistry.

